# Role of EPAC in cAMP-Mediated Actions in Adrenocortical Cells

**DOI:** 10.3389/fendo.2016.00063

**Published:** 2016-06-13

**Authors:** Aurélia E. Lewis, Reidun Aesoy, Marit Bakke

**Affiliations:** ^1^Department of Molecular Biology, University of Bergen, Bergen, Norway; ^2^Department of Biomedicine, University of Bergen, Bergen, Norway

**Keywords:** ACTH, steroidogenesis, cyclic AMP, EPAC, HPA axis, adrenal glands, adrenal cortex hormones

## Abstract

Adrenocorticotropic hormone regulates adrenal steroidogenesis mainly via the intracellular signaling molecule cAMP. The effects of cAMP are principally relayed by activating protein kinase A (PKA) and the more recently discovered exchange proteins directly activated by cAMP 1 and 2 (EPAC1 and EPAC2). While the intracellular roles of PKA have been extensively studied in steroidogenic tissues, those of EPACs are only emerging. EPAC1 and EPAC2 are encoded by the genes *RAPGEF3* and *RAPGEF4*, respectively. Whereas EPAC1 is ubiquitously expressed, the expression of EPAC2 is more restricted, and typically found in endocrine tissues. Alternative promoter usage of *RAPGEF4* gives rise to three different isoforms of EPAC2 that vary in their N-termini (EPAC2A, EPAC2B, and EPAC2C) and that exhibit distinct expression patterns. EPAC2A is expressed in the brain and pancreas, EPAC2B in steroidogenic cells of the adrenal gland and testis, and EPAC2C has until now only been found in the liver. In this review, we discuss current knowledge on EPAC expression and function with focus on the known roles of EPAC in adrenal gland physiology.

## Introduction

In response to stress, the hypothalamic–pituitary–adrenal (HPA) axis is activated. Parvocellular neurons, located in the paraventricular nucleus of the hypothalamus release corticotrophin-releasing factor (CRF), which is transported to the anterior pituitary where it stimulates the corticotropes by binding to type I CRF receptors (1). In response to CRF, these cells release the prohormone pro-opiomelanocortin (POMC) (2). Through posttranscriptional modifications, the inert POMC is converted to biologically active peptides, including adrenocorticotropic hormone (ACTH) (3). ACTH enters the systemic circulation, binds to specific receptors located on the surface of adrenocortical cells, and stimulates the production of adrenocorticosteroid hormones, including cortisol, aldosterone, and adrenal androgens (4). Steroid hormones are produced from the same precursor, cholesterol, by a set of cytochrome P450 steroid hydroxylases (CYP11A1, CYP11B1 and CYP11B2, CYP17 and CYP21) and the steroid dehydrogenase 3βHSD (5). The enzymes are differentially expressed in the three zones of the adrenal cortex (zona glomerulosa, zona fasciculata, and zona reticularis) giving rise to zone-specific hormone production. In humans, the primary source of cholesterol for steroid hormone production is low density lipoprotein (LDL), which is imported via the LDL receptor (LDLR) from the blood stream. Once cholesterol enters the cell, hormone-sensitive lipase (HSL) converts it to free cholesterol substrate (6). Free cholesterol is then delivered to the inner mitochondrial membrane by the actions of steroidogenic acute regulatory protein (StAR) and cholesterol-binding proteins. CYP11A1 (or P450 cholesterol side chain cleavage) catalyses the first and rate-limiting enzymatic step in the biosynthesis of all steroid hormones, the conversion of cholesterol to pregnenolone (7, 8). Pregnenolone can be further converted into different hormone intermediates in the endoplasmic reticulum and the final production of cortisol and aldosterone occurs in mitochondria within the zona fasciculata and zona glomerulosa, respectively (5).

**Figure 1:**
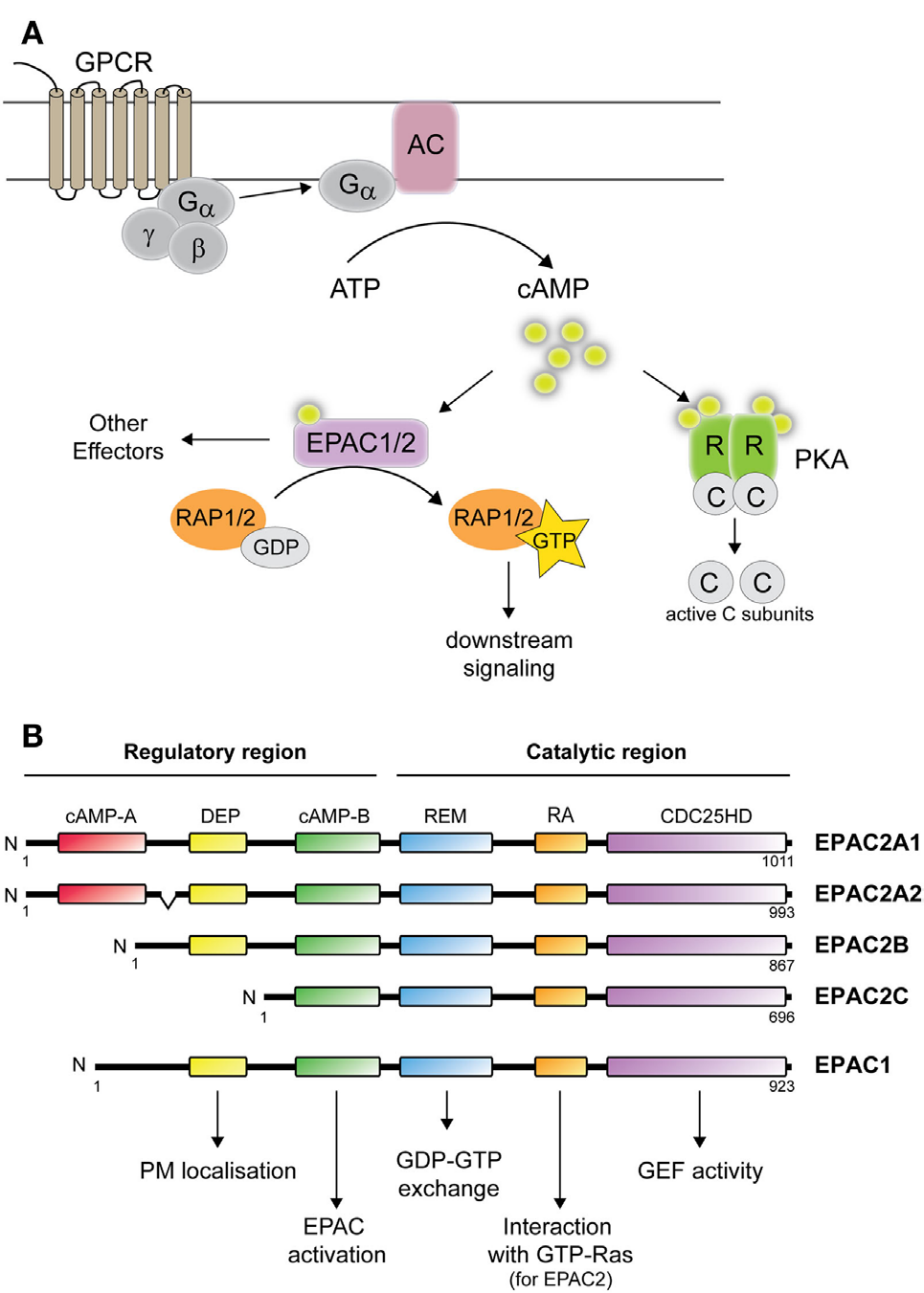
**cAMP-mediated signaling and EPAC1 and 2 isoforms**. **(A)** cAMP signaling: following ligand binding of G-protein-coupled receptor, the membrane bound adenylate cyclase (AC) is activated, and generate cAMP from ATP. cAMP subsequently activates PKA and/or EPAC1/2. Binding of cAMP to PKA causes the release of the catalytic subunits, which phosphorylate a variety of targets. Binding of cAMP to EPAC leads to guanosine diphosphate (GDP) to guanine triphosphate (GTP) exchange on Rap1 or Rap2. **(B)** Domain structure illustration of EPAC1 and EPAC2 isoforms consisting of: cAMP-binding domains A and B, disheveled, Egl-10, pleckstrin (DEP) domain, Ras-exchange motif (REM), Ras association (RA) domain, and CDC25-homology domain (HD). The protein structure of EPAC2A2 is shown in accordance to EPAC2A1. Functional roles of each domain are also indicated. PM, plasma membrane; GEF, guanine nucleotide exchange factor activity.

In the adrenal cortex, ACTH coordinates the biosynthesis of steroid hormones via the second messenger cAMP. In response to ACTH binding to its receptor, conformational changes induce the release of G-proteins, which then activate membrane bound adenylyl cyclases (ACs). Upon activation, ACs generates cAMP from ATP (Figure 1A). cAMP relays ACTH-mediated functions via the activation of the serine–threonine kinase cAMP-dependent protein kinase A (PKA) or the exchange proteins directly activated by cAMP (EPAC1 and 2 also named cAMP-regulated guanine nucleotide exchange factors (cAMP-GEFs) I and II). Following cAMP activation, PKA and EPAC transmit signals differently. PKA phosphorylates numerous substrates, while EPACs act as guanine exchange factors (GEFs) catalyzing the conversion of the small GTPases Rap1 and Rap2 from an inactive (GDP-bound) to active form [guanine triphosphate (GTP)-bound] (9, 10). While cAMP signaling by PKA in steroidogenic cells has been intensely investigated, the roles of EPAC are only beginning to emerge. This review summarizes our current knowledge of EPAC2 in tissues of the hypothalamus–pituitary–adrenal axis.

## Structure and Function of EPAC Isoforms

The identification of EPACs was reported in 1998 by two separate groups using different approaches. EPAC1 was discovered by de Rooij and colleagues in a database search for proteins containing cAMP-binding domains with the ultimate goal to explain the PKA-independent cAMP-induced activation of Rap1 (9). The same year, Kawasaki et al. identified both EPAC1 and EPAC2 in a screen for brain-specific genes with cAMP-binding motifs (11). In recent years, it has become evident that the EPAC proteins play essential roles in many biological processes, in some where EPAC and PKA collaborate to achieve a common biological response and in some where the two cAMP effectors have separate functions [reviewed in Ref. (12–14)].

### Expression of EPAC Isoforms

The EPAC proteins are encoded by two different genes: *RAPGEF3* (EPAC1) and *RAPGEF4* (EPAC2), which both give rise to multiple transcripts. Three transcripts are produced from *RAPGEF3*, but only variant 1, encoding EPAC1, has been studied (13). EPAC1 is relatively ubiquitously expressed (9, 11). Specific parts of the brain, thyroid gland, proximal tubules of the kidney, ovary, and skeletal muscle express the highest levels of EPAC1, but lower levels of EPAC1 have been found in virtually all tissues examined, as well as in hematopoietic cells (15). Studies on murine tissues suggest that EPAC1, as well as EPAC2, mRNA expression is also regulated at different stages of development in embryos and after birth (16).

At the transcript level, four different isoforms arise from *RAPGEF4*, termed EPAC2A1, EPAC2A2, EPAC2B, and EPAC2C (Figure 1B) (17–20). The EPAC2A2 transcript might be specifically expressed in the brain (20), but the existence of a corresponding protein is yet to be demonstrated. Thus, potential biological roles for this isoform remain unknown. By contrast, the three other mRNAs are known to give rise to the proteins EPAC2A, EPAC2B, and EPAC2C. EPAC2A was the initial EPAC2 isoform identified (11), and is expressed predominantly in brain (with high levels in the cerebral cortex, hippocampus, habenula, cerebellum, and hypothalamus), pituitary, and endocrine pancreas (11, 17, 20). The EPAC2B isoform was identified by the group of Dr. Seino during their efforts in developing an EPAC2 knockout model (19). While confirming deletion of EPAC2A, they discovered the presence of a shorter transcript in the adrenal gland that lacked the N-terminal cAMP-binding domain [(19); Figure 1B]. Until now, EPAC2B expression has only been demonstrated in the adrenal gland (19–21), in the Leydig cell-derived cell line MA10 (21) and in endocrine pancreas (20). The physiological roles of EPAC2B appear to diverge from those of EPAC2A since EPAC2B is not able to substitute for EPAC2A in cellular assays monitoring insulin secretion (19). The shortest EPAC2 isoform, EPAC2C, was also identified by the group of Dr. Seino (17, 18) and has so far only been found in the liver, presumably solely in hepatocytes (18, 20). The strict tissue-specific expression of the different EPAC2 isoforms is controlled, at least in part, by DNA methylation. Detailed analyses of the EPAC2 gene have led to the identification of alternative promoters for the different isoforms (18, 20) and bisulfite sequencing demonstrated that the methylation status of the different promoters nearly perfectly mimics their activity and the expression pattern of the corresponding isoform (20).

### Structure and Activation of EPAC Proteins

EPACs are multidomain proteins consisting of two main parts, i.e., an N-terminal regulatory region and a C-terminal catalytic region (Figure 1B). The regulatory region is built up by a cAMP-binding domain and a disheveled, Egl-10, pleckstrin (DEP) domain. EPAC2A contains two cAMP-binding domains, cAMP-A and cAMP-B, while EPAC1 has only one such domain. The domain structure of EPAC2B is similar to EPAC1 and lacks the first cAMP-A binding domain, while EPAC2C lacks both the cAMP-A and DEP domains (18, 19). The N-terminal cAMP-A domain in EPAC2A binds cAMP with low affinity and is not believed to be important for cAMP-induced activation (10). Instead, this domain appears to be important for the localization of EPAC2A near the plasma membrane (19). The DEP domain, by its ability to interact with phosphatidic acid, is also important in targeting EPAC to the plasma membrane upon activation by cAMP (22). The catalytic region consists of a CDC25 homology domain (CDC25HD) that catalyzes Rap1 activation, a Ras-exchange motif (REM) domain, and a Ras association (RA) domain (23). The regulatory regions of EPAC1 and EPAC2 function as inhibitors of the C-terminal GEF domain in the absence of cAMP. Binding of cAMP induces a conformational change that opens the catalytic CDC25HD domain from auto-inhibitory restraints and thereby permits GTP loading of Rap (10, 24–26). Both EPAC1 and EPAC2 contain potential RA domains, but only EPAC2 has been shown to interact with Ras-GTP, which contributes in recruiting EPAC2 to the plasma membrane. The enrichment of EPAC2 on the membrane through Ras binding is crucial for EPAC2-mediated Rap1 activation (27, 28). In addition to the plasma membrane, other subcellular localizations have been observed, such as the perinuclear region, nuclear membranes, and mitochondria for EPAC1 [reviewed in Ref. (29)] and the Golgi apparatus and the nucleus for EPAC2B (21).

### Physiological Roles of EPAC

EPACs regulate a multitude of cAMP-mediated cellular processes in many different tissues [extensively reviewed in Ref. (23)], including the formation of cell–cell adhesion (24, 30–33), cell proliferation (34, 35), differentiation (36, 37), cell survival (38), ion channels regulation (39–41), and Ca^2+^-mediated signaling (23, 42, 43). The development of EPAC knockout mice models has led to a better insight into the biological functions of these proteins. In spite of the involvement of EPAC in multiple cellular pathways, mice lacking EPAC1, EPAC2, or both EPAC1 and EPAC2 do not show gross developmental or reproductive abnormalities. However recent studies have revealed that EPAC1−/− and EPAC2−/− mice display various phenotypes in response to stress or other challenges. For example, mice lacking EPAC1 or EPAC2 exhibit impaired glucose tolerance and dysfunctional insulin secretion after glucose challenge when compared with their wild-type littermates (44–46). Double knockout mice of both EPACs in the forebrain showed defects in long-term potentiation, spatial learning, and social interactions (47), whereas knocking out only EPAC2 is sufficient to induce social interactions impairment (48). Loss of EPAC2 also causes defects in memory retrieval in a fear condition paradigm (49). Interestingly, single nucleotide polymorphisms within the gene encoding EPAC2 have been linked to autism. Screening of 48 autistic individuals for mutations in the RAPGEF4 gene showed that four rare missense mutations may be a cause of autism (50). In spine synapses, these mutations alter the protein function of EPAC2 by affecting its Rap-GEF activity, the synaptic protein distribution and spine morphology (51). Activation of EPAC2 results in shrinkage of dendritic spine size as well as increased motility and turnover of the spines, thereby contributing to the plasticity of brain circuits (51, 52). Mice lacking EPAC1 or EPAC2 also present with phenotypes in the heart. Deletion of EPAC1 causes a mild decrease in basal cardiac functions, but more interestingly protects mice hearts from various stressors, such as arrhythmogenic stress (53). However, partly in contrast to this study, deletion of EPAC1 was reported to have no effect in cardiac function (54). Instead, loss of EPAC2 was shown to protect against β-adrenergic receptor-dependent arrhythmia (54). Most of the biological functions aforementioned have been attributed retrospectively to EPAC1 and EPAC2A due to their expression pattern. The roles of EPAC2B and EPAC2C, which were discovered later, are overall less studied. The potential roles of EPAC2B are discussed in chapter 2. In the liver, EPAC2C has been shown to suppress apoptosis and iNOS expression and activity in hepatocytes (55). EPAC2C may also control bile acid-stimulated canalicular formation in the liver (56).

## EPAC in the HPA Axis

EPAC2A is expressed in the hypothalamus and pituitary gland and EPAC2B in the adrenal gland. cAMP is known to be an essential regulator at all levels in the HPA axis (57) and, here, we review how EPAC2 is emerging to contribute to cAMP-mediated actions.

### EPAC in the Hypothalamus and the Pituitary

In response to various stress factors, the expression of CRF is stimulated in the PVN of the hypothalamus. CRF synthesis is dependent in part upon the neuropeptide pituitary adenylate cyclase-activating polypeptide (PACAP) and a subsequent elevation of cAMP (57, 58). So far, only PKA-dependent signaling has been reported to relay the stimulatory effect of cAMP on CRF expression. PKA inhibition was indeed shown to prevent binding of cAMP response element (CRE)-binding protein (CREB) on the CRF gene promoter and to inhibit transcriptional activation (59). EPAC has not been studied in PACAP-mediated actions in the PVN. However, EPAC was shown to mediate the effects of PACAP on long-term depression of synaptic transmission in the hippocampus through Rap in murine hippocampal slices (60), and in mice deleted for both Epac1 and Epac2 (47). These studies may, therefore, suggest a potential contribution of EPAC in PACAP-responsive PVN. In the suprachiasmatic nuclei of the hypothalamus, EPAC has been associated with leptin signaling (61) and the regulation of factors involved in setting circadian rhythms in Ref. (62). In noradrenergic neurons isolated from locus coeruleus (LC) in culture, EPAC, but not PKA, was shown to be involved in mediating the actions of cAMP (63). Thus, upon CRF binding to type-1 CRF receptor, LC neurons differentiate into norepinephrine-producing neurons via the activation of cAMP–EPAC–ERK/MAPK pathway, by potentiating brain-derived neurotrophic factor-stimulated synaptic plasticity via tyrosine kinase B signaling (63). Since LC neurons innervate PVN neurons, EPAC may, hence, indirectly stimulate the secretion of CRF from the hypothalamus [reviewed in Ref. (64)].

EPAC2A is also the dominant isoform expressed in the pituitary (19), but we still have very limited information about the potential roles of EPAC2 in this gland. Experiments in AtT20 pituitary cells demonstrated that EPAC, presumably EPAC2A, acts as a mediator of CRF_1_-induced signaling in corticotropes (65) and in HEK-293 overexpressing CRH-R2β cells (66). Upon activation of the CRH receptor, EPAC2A is involved in cAMP-mediated induction of ERK signaling (65), a pathway previously reported to induce POMC transcription in a PKA-independent manner (67). In addition, CRF receptor activation via Gq is known to signal to phospholipase Cɛ (PLCɛ) and, hence, inositol (1,4,5) triphosphate (IP3) to induce calcium stores mobilization that contributes to ACTH secretion (1, 2, 68). Considering that the activation of PLCɛ by EPAC–Rap2 has been reported to activate Ca^2+^ release and the secretion of hormones in different tissues (23), EPAC may also contribute to the secretion of ACTH in corticotropes.

### EPAC in Adrenal Physiology

#### Role of EPAC versus PKA in Steroidogenesis

In the adrenal cortex, PKA is undoubtedly the major mediator by which cAMP regulates steroidogenesis (69–71). Once PKA is activated, both an acute and a chronic response occur, which contribute to increased steroid hormone synthesis. During the acute response, PKA phosphorylates HSL, which converts cholesterol esters to free cholesterol. This rapid response also involves an increase in StAR, which facilitates the movement of cholesterol to the inner mitochondrial membrane where the rate-limiting enzyme CYP11A1 resides (72). The chronic response corresponds to the transcriptional activation of all the other steroidogenic enzymes (73, 74). In a study by Schimmer et al., using microarray technology to investigate the effects of ACTH in mouse adrenal Y1 cells, the involvement of PKA was shown to account for up to 60% of the effects of ACTH on transcription, while only 6% could be assigned to PKC (70). This study clearly validated the dominant role of PKA in steroidogenesis, but left about 34% of the ACTH effects to be independent of PKA and PKC. In addition, another study had pointed to the importance of cAMP signaling, mediated independently of PKA, for aldosterone production in the adrenal zona glomerulosa (75). These findings suggest a role for EPAC in the regulation of adrenal function. The specific expression of the EPAC2B isoform in steroidogenic cells (19, 21) also points to roles for EPAC-dependent signaling in these cells. We, therefore, systematically assessed the involvement of PKA versus EPAC in steroidogenesis using cell permeable cAMP analogs specific for PKA and EPAC1/2 (N6-benzoyl-cAMP and 8-p-chlorophenylthio-2-*O*-methyl-cAMP) in adrenocortical cell lines (21). Our study demonstrated that PKA, and not EPAC2B, is the essential cAMP-induced regulator of factors involved in steroid hormone production (such as StAR, CYP11A1, and CYP17) as well as for the biosynthesis of cortisol and aldosterone. The role of EPAC2 was also studied in bovine zona fasciculata, expressing high levels of EPAC2 mRNA (76), using the same EPAC-specific cAMP analog (77). Although this analog induced cortisol biosynthesis, a non-hydrolyzable EPAC activator had no effect. The study concluded that metabolites of the hydrolyzable EPAC-specific analog induced the increase in cortisol observed in a cAMP-independent manner. Although seemingly opposite results were obtained with the same EPAC activating compound, these two studies indicate that EPAC is not important for cortisol production. cAMP rapidly induces the transcription factor nerve growth factor-induced clone B (NGFI-B), a regulator of several steroid hydroxylase genes (78–80). In adrenocortical cells in culture, we also found that NGFI-B-induction by cAMP is mediated by PKA and not by EPAC (21). Current investigations on HPA axis regulation in mouse knockout models will provide insights into the potential roles for EPAC1/2 in this neuroendocrine system.

#### EPAC2B Contributes to Cytoskeletal Remodeling in the Adrenal Cortex

In adrenocortical cells in culture, cAMP characteristically induces changes in cell shape and a concomitant reorganization of F-actin microfilaments [reviewed in Ref. (81)]. Furthermore, cytoskeletal reorganization has been shown to contribute to steroidogenic hormone production by allowing the correct positioning of lipid droplets, the ER and mitochondria where cholesterol and its metabolites are transported and metabolized (82–84). While using PKA- and EPAC-specific agonists to study their effect on steroidogenesis in adrenocortical cell lines, we observed that the activation of both cAMP effectors contributed to cell rounding and the reorganization of F-actin fibers (21). Considering that PKA activation contributes to the regulation and expression of many enzymes necessary for steroidogenesis, the additional effect mediated by PKA on F-actin remodeling would, hence, correlate well with enzymatic outputs. By contrast, the effects of EPAC on F-actin remodeling are not correlated to steroidogenesis and may, therefore, contribute to other aspects of adrenal physiology. In line with this, we also found that activation of EPAC2B induced a marked decrease in migration (21). This finding implies that EPAC2B plays a role in cell motility and this suggests wider implications, such as adrenal cancer cell invasion. Although EPAC2 has so far not been implicated in cancer development, several studies have demonstrated roles, albeit contradictory, for EPAC1 in cell migration and metastasis (85). The molecular mechanisms implicated include, at least, an increase of Ca^2+^ release mediated by PLC-IP3 promoting actin remodelling and cell migration of melanoma cells (86) as well as integrin activation important for cell migration and metastasis of pancreatic cancer cells (87). EPAC2B may, hence, act in the same way as EPAC1 in the adrenal gland.

## Conclusion

Since the discovery of EPACs in 1998, our understanding of cAMP-induced signaling and its roles in physiological processes has changed dramatically. Important initial *in vitro* studies on EPAC paved the way for current phenotypic analyses of genetic mouse models lacking EPAC in single or double knockouts. Based on these gene knockout models an important picture has emerged, namely that although deletion of EPAC does not cause gross defects in mice kept at standard protected conditions in the animal facility, exposure to stressful situations provoke significant phenotypes. EPAC2 is expressed along the HPA axis, and it is interesting to note that whereas the hypothalamus and pituitary specifically express EPAC2A, the adrenal cortex expresses solely the EPAC2B isoform. While EPAC has been shown to mediate potential roles in the hypothalamus, putative functions in the PVN are yet to be determined. At the hypophyseal level, EPAC2A has been implicated in the regulation of POMC expression, and in the adrenal cortex, EPAC2B affects the migration of adrenocortical cells in culture. The generation of spatial and temporal conditional gene knockout models is now required to pinpoint the specific roles of the different EPAC isoforms during development and adult life. Moreover, the ongoing efforts to develop isoform-specific agonists and antagonists hold great promise for insights into isoform-specific functions. Such compounds will also be important potential new drugs to treat diseases in which EPAC plays a role. Several studies do indeed suggest that EPACs are promising drug targets (88), giving hope that small molecules targeting EPACs will serve as useful treatments in the future.

## Author Contributions

AEL, RA, and MB wrote parts of the manuscript. AL coordinated editing.

## Conflict of Interest Statement

The authors declare that the research was conducted in the absence of any commercial or financial relationships that could be construed as a potential conflict of interest.
